# Ipsilateral vagotomy to unilaterally ovariectomized pre-pubertal rats modifies compensatory ovarian responses

**DOI:** 10.1186/1477-7827-5-24

**Published:** 2007-06-13

**Authors:** Leticia Morales, Beatriz Ricardo, Adán Bolaños, Roberto Chavira, Roberto Domínguez

**Affiliations:** 1 Biology of Reproduction Research Unit. Physiology of Reproduction Laboratory FES Zaragoza. UNAM. AP 9-020, CP 15000, México, DF., México; 2 Instituto Nacional de Ciencias Médicas y de la Nutrición "Salvador Zubirán" México

## Abstract

The present study evaluates the participation of the vagus nerve in pre-pubertal rats with unilateral ovariectomy on puberty onset, and on progesterone, testosterone and estradiol serum levels, and the compensatory responses of the ovary. Unilateral vagotomy did not modify the onset of puberty in unilaterally ovariectomized rats. Ovulation rates of animals with the left vagus nerve sectioned and the left ovary in-situ was lower than in rats with only unilateral ovariectomy. Sectioning the left vagus to 32-day old rats with the left ovary in-situ resulted in lower compensatory ovarian hypertrophy than in rats with right unilateral ovariectomy. Twenty-eight or 32-day old animals with sectioning of the right vagus nerve and the right ovary in situ showed higher compensatory ovulation. Twenty-eight -day old rats with the right ovary in situ had higher progesterone and testosterone levels than animals of the same age with the left ovary in-situ. Compared to animals with the right ovary in situ, animals treated at 32-days of age, sectioning the ipsi-lateral vagus nerve resulted in higher progesterone levels. Higher progesterone levels were observed in 28- and 32 days old rats with the left ovary in situ and left vagus nerve sectioned. Thirty-two day old animals with the right ovary in situ and right vagus nerve sectioned had higher progesterone levels than rats of the same age with the left ovary in situ and left vagus nerve sectioned. Left vagotomy to 28-day old rats with the left ovary in situ resulted in higher testosterone levels, a reverse response to that observed in animals with sectioning of the right vagus and the right ovary in situ. Thirty-two day old rats with the left ovary in situ and left vagus nerve sectioned showed lower testosterone levels than animals without vagotomy and with the left ovary in situ.

Twenty-eight -day old animals with the left vagus sectioned and left ovary in situ had lower estradiol serum levels than rats without unilateral vagotomy, a response similar to that observed in 32-day old rats with the right ovary in situ and right vagus nerve sectioned.

Present results suggest an asymmetric regulation of steroid hormones secretion by the vagus nerve innervations in animals with unilateral ovariectomy, and those differences in testosterone serum levels observed are associated to the ovary remaining in-situ, vagal innervation and age when the animals were treated.

## Background

The vagus nerve is one of the neural pathways involved in regulating ovarian functions [1-9] Gerendai and collaborator's provided the first morphological evidence of a multi-synaptic neural pathway between the ovary and the central nervous system (CNS), showing that the vagus nerve forms part of such neural connection [6,7,10]. Unilateral ovariectomy (ULO) is an experimental tool used to analyze the existence of functional asymmetry between the ovaries. In the rat, removal of one ovary results in increases of ovarian weight (compensatory ovarian hypertrophy [COH]) and in the number of ova shed (compensatory ovulation, [CO]) by the in situ ovary [1,11,12]. COH results in the rise of the follicle population and a decrease in the rate of follicular atresia [13]. These effects have been explained as resulting from lower systemic ovarian steroid levels that partially remove the negative feedback over the hypothalamic-pituitary axis, triggering a higher release of gonadotropins [14,15].

Previously, we showed that increasing and maintaining elevated levels of follicle stimulating hormone (FSH) in serum does not always result in ovarian weight increase and higher ovulatory responses by the in situ ovary [16]. Modifications in FSH and luteinizing hormone (LH) serum levels, as well as changes in the number of ovulatory follicles induced by extirpating one ovary depend on the day of the estrous cycle when surgery is performed and on the time elapsed between treatment and autopsy [16].

At present, ovarian compensatory responses are not only explained by hormonal variations. Burden and Lawrence [1] suggested that the ovarian innervation modulates ovarian functions. Ample experimental evidence supporting the idea that ovarian functions are modulated by at least two different neural pathways, the noradrenergic system (via the superior ovarian nerve and the ovarian plexus) and the parasympathetic system (via the vagus nerve), have been published [6,7,9,17-23].

Studies on the participation of the vagus nerves in regulating ovarian functions have been performed mainly in adult animals. These studies have shown that bilateral sectioning of the vagus nerve results in a reduction in the number of ova shed [24]; that the length of the estrous cycle increases when bilateral sectioning of the vagus nerves is performed during the days of estrous or pro-estrous [25]; and that bilateral vagotomy blocks both, pseudo-pregnancy induced through cervical stimulation [26], and the COH in rats with ULO [1,27]. Furthermore, bilateral vagotomy did not change the ovulation rates in animals with only ULO [28].

In adult rats, the effects of unilateral vagotomy on ovarian functions in rats depend on which vagus nerve is sectioned. Rats with right ULO (left ovary in situ) and the left vagus nerve sectioned showed a significant increase in ovulation rates and number of ova shed by ovulating animals, while rats with left ULO (right ovary in situ) and right vagotomy showed a decrease in ovulation rates and number of ova shed [28].

The participation of the vagus nerve in regulating ovarian functions in the pre-pubertal rat is poorly understood. Ojeda et al. [9] showed that bilateral abdominal vagotomy resulted in a marked delay of puberty onset. Also, when the ovaries of bilateral vagotomized rats were kept *in vitro*, estradiol and progesterone secretion, triggered by exposure to human chorionic gonadotropins (hCG), were markedly depressed in ovaries obtained from rats with abdominal vagotomy.

In a previous study we showed that the effects of unilateral or bilateral vagotomy to 24 or 28-day old rats are different. Unilateral or bilateral vagotomy performed to 24-day old rats did not modify ovulation rates or the number of ova shed. In turn, compared to untouched – control- animals, bilateral vagotomy performed to 28-days old rats resulted in a significant increase in the number of ova shed by ovulating animals. Bilateral vagotomy or unilateral vagotomy of the right branch to 28-day old rats resulted in lower estradiol levels and a delay in puberty onset (expressed by noticeable vaginal opening), while unilateral vagotomy of the left branch did not cause the same effects. In turn, unilateral vagotomy to 24-day old rats did not modify progesterone levels, while bilateral vagotomy resulted in a significant increase of progesterone serum levels. These results were interpreted as indicating that the innervations reaching the ovary via the vagus nerve regulate, in an inhibitory way, the onset of puberty, and that secretion of ovarian hormones depends on the remaining vagus nerve and the age when animals are treated [8].

Based on the different responses to unilateral and bilateral sectioning of the vagus nerve by pre-pubertal and adult rats, the present study aims to analyze the effects, in unilateral ovariectomized rats, of unilaterally sectioning the vagus nerve of the in situ ovary on puberty onset, and CO, COH and ovarian hormones levels. Animals were sacrificed the day of first vaginal opening (puberty).

## Materials and methods

All experiments were carried out in strict accordance with the Mexican Law of Animal Treatment and Protection Guidelines. The Committee of the Facultad de Estudios Superiores Zaragoza approved the experimental protocols.

The study was performed using pre-pubertal female rats of the CIIZ-V strain from our own breeding stock. Animals were maintained under controlled lighting conditions (lights on from 05:00 to 19:00 h); with free access to rat chow and tap water. Surgery procedures were performed under ether anesthesia between 10:00 and 12:00 h. Control group and treated rats were inspected daily for vaginal opening (VO). Daily vaginal smears were obtained after VO was observed.

In previous studies we showed that the participation of the superior ovarian nerve in 28 and 32 days old rats is different [21,22]. Then, 28 or 32 day old animals were randomly assigned to one of the following experimental groups:

*Untreated control*: A group of 15 untreated animals were killed the day of first vaginal estrus.

*Laparotomy (sham-operated group)*: eleven 28-day old and twelve 32-day old rats were anesthetized, a ventral laparotomy was performed and the wound was subsequently sealed.

*Unilateral ovariectomy (ULO)*: Animals were anesthetized and laparotomized, and the right (ten 28-day and nine 32-day old) or left (thirteen 28-day old and sixteen 32-day old) ovary extirpated. The wound was subsequently sealed.

*Ipsilateral sectioning of the vagus nerve followed by unilateral ovariectomy: *Sectioning the vagus nerve was performed following previously described methodology [25]. In brief, animals were anesthetized and laparotomized, the liver was reflected, the esophagus exposed, and ventral (left) or dorsal (right) vagus was cut with fine forceps. Immediately after sectioning the vagus nerve, ULO was performed: the left ovary was removed from animals with the right vagus nerve sectioned, and the right ovary from rats with the left vagus nerve sectioned. The wound was subsequently sealed and the animals returned to their group cage.

### Autopsy procedures

Animals were killed by decapitation, between 10 am and noon, on the first day of vaginal estrus (VE). The blood from the trunk was collected, allowed to clot, and centrifuged during 15 min at 3,000 rpm. The serum was stored at -20°C, until progesterone, testosterone and estradiol levels were measured. At autopsy, the oviducts were dissected and the number of ova counted with the aid of a dissecting microscope. Ovaries were removed, dissected, and weighed in a precision balance.

CO and COH were calculated as follows:

CO = [(NO – MNO)/MNO] × 100

Where NO is the number ova shed by the ovary in situ and MNO is the mean number of ova shed by the respective ovary in the sham operated group.

COH = [(WO – MWO)/MWO] × 100

Where WO is the weight of the ovary in situ and MWO is the mean weight of the respective ovary from the sham operated group.

### Hormone measurement

Estradiol, testosterone and progesterone serum concentrations were measured by radioimmunoassay (RIA), using kits purchased from Diagnostic Products (Los Angeles, CA, USA). The intra and inter-assay coefficients of variation were 8.35% and 9.45% for progesterone, 8.12% and 9.28% for estradiol, and 9.65% and 10.2% for testosterone. Progesterone values are expressed as nanograms per milliliter (ng/ml), and estradiol and testosterone as picograms per milliliter (pg/ml).

### Statistical analysis

Data on ovaries' weight, and progesterone, testosterone and estradiol concentrations, were analyzed by multivariate analysis of variance (MANOVA), followed by Tukey's test. The age of VO, first ovulation, CO and COH were analyzed by Kruskal-Wallis test, followed by Mann-Whitney U-test. Ovulation rate (number of ovulating animals/number of treated one) was analyzed by Fisher's exact probability test or the Chi square test. A P-value of less than 0.05 was considered significant.

## Results

Compared to untreated rats, sham operation performed to 28 or 32-day old rats resulted in a significant delay in the age of VO and first VE. The effects of ULO were compared with their respective sham operation group. Similar ages in VO and first VE were observed between animals with ULO (left or right) and their respective sham operated group (Table 1). The COH and CO was similar in animals with the left or right ovary *in situ *(COH 28 days-treated rats: left ovary 48 ± 10 vs. right ovary: 67 ± 14.4; 32 days-treated rats: left ovary 50.8 ± 6.3 vs. right ovary: 52.7 ± 11.2; CO 28 days-treated rats: left ovary 76.4 ± 6.6 vs. right ovary: 86.7 ± 10; 32 days-treated rats: left ovary 77.8 ± 5.1 vs. right ovary: 84.6 ± 5.2, non significant;).

**Table 1:** Means ± SEM of day of vaginal opening and first vaginal estrus in untreated animal, sham operated and ULO rats treated at 28 or 32 days of age.

Group	N	Age of treatment	In situ ovary	Vaginal opening	First vaginal estrus
Untreated	15		Both	35.8 ± 0.5	36.7 ± 0.6
					
Sham	13	28 days	Both	38.7 ± 0.8*	40.1 ± 0.9*
ULO	10	28 days	Left	38.7 ± 0.7	40.5 ± 1.1
ULO	13	28 days	Right	39.8 ± 0.3	41.8 ± 0.6
					
Sham	8	32 days	Both	38.2 ± 0.5*	40.7 ± 1.1*
ULO	9	32 days	Left	39.2 ± 0.7	41.0 ± 0.9
ULO	16	32 days	Right	38.6 ± 0.6	39.9 ± 0.6

p < 0.05 vs untreated animal group (Student's test).

### Ovulation rate in rats with the ovary extirpated immediately after sectioning the ipsilateral vagus nerve

**Figure 1:**
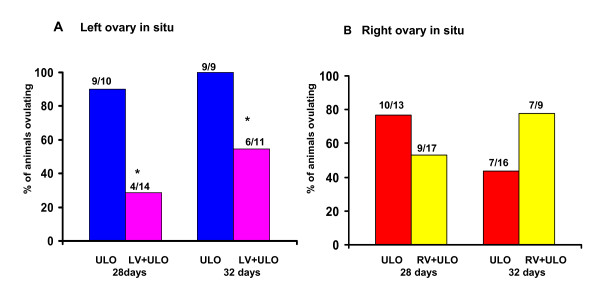
**Ovulation rates in rats with section of the vagus nerve and unilateral ovariectomy**. Ovulation rate (number of animals ovulating/number of treated animals) in rats with left (LV) or right (RV) sectioning of the vagus nerve and unilateral ovariectomy (ULO), left ovary *in situ *(A) or right ovary *in situ *(B). Animals were sacrificed on the morning of the day of first vaginal estrus. *p < 0.05 vs ULO (Fisher test)

Compared to animals with ULO, extirpating the right ovary to 28 or 32-day old rats with left vagotomy resulted in lower ovulation rates (Figure 1A); while the reverse treatment, extirpating the left ovary to rats with right vagotomy, did not modify ovulation rates (Figure 1B).

### Effects of unilateral ovariectomy on compensatory response in rats with ipsilateral vagotomy

#### Compensatory ovarian hypertrophy

**Figure 2:**
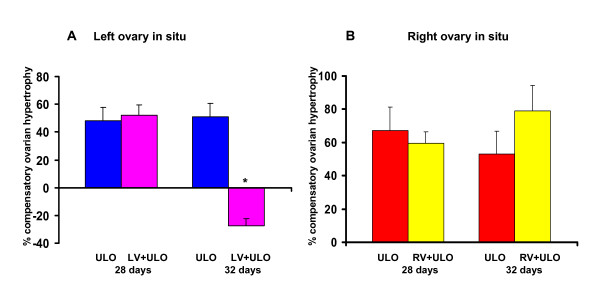
**Compensatory ovarian hypertrophy in rats with section of the vagus nerve and unilateral ovariectomy**. Mean ± mean standard error, compensatory ovarian hypertrophy in rats with left (LV) or right (RV) sectioning of the vagus nerve and unilateral ovariectomy (UO), left ovary *in situ *(A) or right ovary *in situ *(B). Animals were sacrificed at first estrus. * p < 0.05 vs ULO left ovary in situ (U-Mann Whitney test)

Left vagotomy performed to 32-day old rats with the left ovary in situ showed lower COH than rats without unilateral vagotomy. Such effect was not observed in 28-day old treated rats (Figure 2A). Sectioning the right vagus nerve before extirpating the left ovary did not modify COH (Figure 2B).

#### Compensatory ovulation

Extirpating the right ovary of rats with left vagotomy did not modify CO (Figure 3A), but left ULO right vagotomy resulted in significantly higher CO than in ULO rats (Figure to rats with 3B).

### Effects of unilateral ovariectomy on hormone serum levels in rats with ipsilateral vagotomy

**Figure 3:**
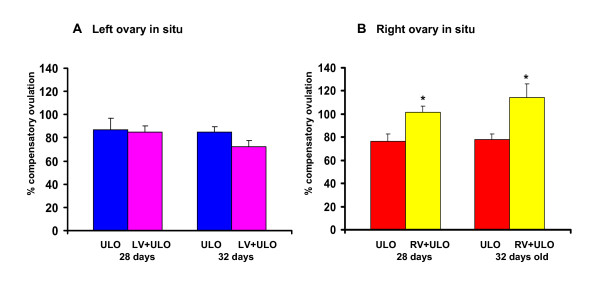
**Compensatory ovulation in rats with section of the vagus nerve and unilateral ovariectomy**. Detailed legend: Mean ± mean standard error of compensatory ovulation in rats with left (LV) or right (RV) sectioning of the vagus nerve and unilateral ovariectomy (UO), left ovary *in situ *(A) or right ovary *in situ *(B).. Animals were sacrificed at first estrus. * p < 0.05 vs ULO right ovary in situ (U-Mann Whitney test)

#### Progesterone

No significant differences in progesterone serum levels were observed between sham-operated and control rats (28 days 4.9 ± 0.6; 32 days 4.1 ± 0.6 *vs*. 4.7 ± 0.6, n.s.). Compared to sham-operated animals, ULO did not modify progesterone serum levels when the animals were treated at 32-days of age, while in 28-day old animals progesterone levels were lower than in sham operated ones. Progesterone levels in rats with the right ovary in situ were higher than in animals with the left ovary in situ (Table 2).

**Table 2:** Means ± SEM of progesterone serum levels in sham operated and ULO rats treated at 28 or 32 days of age, sacrificed the day of first vaginal estrous.

Group	N	Age of treatment	In situ ovary	Progesterone (ng/ml)
Sham	13	28 days	Both	4.9 ± 0.6
				
ULO	10	28 days	Left	3.1 ± 0.3*
ULO	13	28 days	Right	6.5 ± 1.0◆
				
Sham	8	32 days	Both	4.1 ± 0.6
ULO	9	32 days	Left	2.7 ± 0.3
ULO	16	32 days	Right	4.2 ± 0.6◆

* p < 0.05 vs. sham ◆ p < 0.05 vs. rats with left ovary in situ, same age of treatment

**Figure 4:**
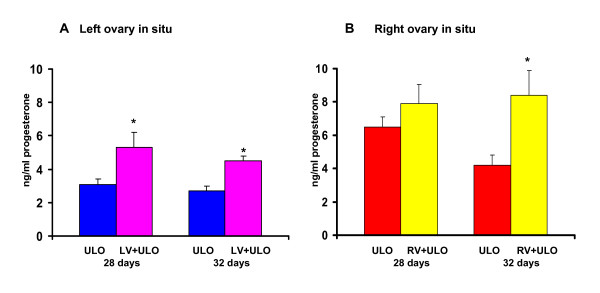
**Progesterone (ng/ml) serum concentration in rats with section of the vagus nerve and unilateral ovariectomy**. Mean ± mean standard error of progesterone serum concentration in rats with left (LV) or right (RV) sectioning of the vagus nerve and unilateral ovariectomy (ULO) the side right (A) or left (B) performed at 28 or 32 day old rats. Animals were sacrificed at first estrus. *p < 0.05 vs. ULO with respective ovary in situ ("t" Student test)

Extirpating the right ovary of 28 or 32-day old rats with left vagotomy resulted in higher progesterone levels than in rats with ULO only (Figure 4A). Extirpating the left ovary of 28 or 32-day old with right vagotomy increased progesterone serum levels, but the increase was significant only in rats treated 32-day old (Figure 4B).

#### Testosterone

Compared to untouched control animals, no significant changes in testosterone serum levels were observed in 28-day old sham-operated animals (31.7 ± 8.4 *vs*. 53.8 ± 7.4, n.s.), while sham-operation performed to 32-day old rats resulted in significantly lower testosterone concentrations (15.3 ± 4.2 *vs*. 53.8 ± 7.4, p < 0.05). Performing right ULO to 28-day old rats resulted in lower testosterone levels compared to sham-operated rats. The same treatment performed to 32-day old animals resulted in an reverse response.

In 28-day old ULO treated rats, testosterone concentration was higher in rats with the right ovary *in situ *than in rats with the left ovary *in situ *(Table 3). Such difference was not observed in rats treated at 32-days old

**Table 3:** Means ± SEM of testosterone (pg/ml) serum levels in sham operated and ULO rats treated at 28 or 32 days of age, sacrificed the day of first vaginal estrous.

Group	N	Age of treatment	In situ ovary	Testosterone (pg/ml)
Sham	13	28 days	both	31.7 ± 8.4
ULO	10	28 days	left	17.9 ± 2.9*
ULO	13	28 days	right	47.7 ± 6.5^◆^
				
Sham	8	32 days	both	15.3 ± 4.2
ULO	9	32 days	left	31.6 ± 5.6*
ULO	16	32 days	right	21.7 ± 2.2

* p < 0.05 vs. sham rats; ^◆ ^p < 0.05 vs. rats with left ovary in situ, same age of treatment.

**Figure 5:**
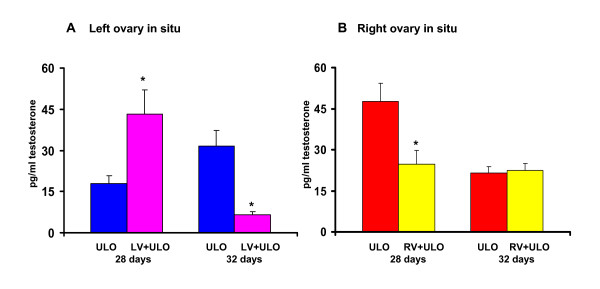
**Testosterone (pg/ml) serum concentration in rats with section of the vagus nerve and unilateral ovariectomy**. Mean ± mean standard error of testosterone serum concentration in rats with left (LV) or right (RV) sectioning of the vagus nerve and unilateral ovariectomy (ULO) the side right (A) or left (B) performed at 28 or 32 days old rats. Animals were sacrificed at first estrus. *p < 0.05 vs. ULO with respective ovary in situ ("t" Student test)

ULO rats, treated at 28 day-old rats, with the left ovary in situ and left vagotomy had higher hormone levels than rats with the left ovary in situ without vagotomy. An reverse response was observed in 32-day old animals with the same treatments (Figure 5A).

Testosterone concentration in 28-day old animals was significantly lower in animals with the right vagotomy and the right ovary in situ than in rats with right ovary in situ without vagotomy. Compared to rats with ULO only, right vagotomy followed by left ULO to 32-day old animals did not modify testosterone serum levels (Figure 5B).

#### Estradiol

Compared to untouched control animals, sham operation to 28 or 32-day old animals resulted in significantly lower estradiol serum levels (28 days: 7.9 ± 0.8; 32 days: 14.7 ± 1.42 *vs*. 37.8 ± 3.5, p < 0.05); therefore, estradiol serum levels in treated animals were compared to the sham-operated group.

Compared to sham-operated animals, ULO performed to 28 or 32-day old animals did not modify estradiol serum levels (Table 4). In animals treated at 32-days of age, the right ovary released more estradiol than the left one.

**Table 4:** Means ± SEM of estradiol serum levels in sham operated and ULO rats treated at 28 or 32 days of age, sacrificed the day of first vaginal estrous.

Group	N	Age of treatment	In situ ovary	Estradiol (pg/ml)
Sham	13	28 days	both	7.9 ± 0.8
ULO	10	28 days	left	10.3 ± 1.0
ULO	13	28 days	right	8.4 ± 0.4
				
Sham	8	32 days	both	14.7 ± 1.4
ULO	9	32 days	left	13.9 ± 1.2*
ULO	16	32 days	right	18.7 ± 1.7*◆

*p < 0.05 vs. rats treated at 28-days of age (Student's *t *test); ◆ p < 0.05 vs. rats with left ovary *in situ*, same age of treatment (Student's *t *test).

**Figure 6:**
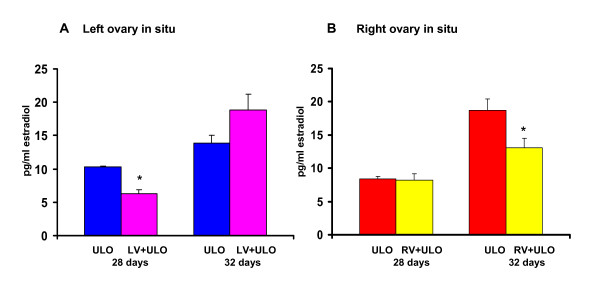
**Estradiol (pg/ml) serum concentration in rats with section of the vagus nerve and unilateral ovariectomy**. Mean ± mean standard error of estradiol serum concentration in rats with left (LV) or right (RV) section of the vagus nerve and unilateral ovariectomy (ULO) performed on 28 or 32-day old rats. Animals were sacrificed at first estrus. *p < 0.05 vs. ULO with respective ovary in situ ("t" Student test)

Left vagotomy followed by right-ULO to 28-days old rats resulted in lower estradiol levels compared to animals with only ULO treatment. Such effects were not observed in animals treated at 32-days of age (Figure 6A).

Compared to ULO treated animals, right vagotomy to 28-day old rats followed by left-ULO did not modify estradiol levels, while the same treatment performed to 32-day old rats in rats with vagotomy resulted in lower estradiol levels (Figure 6B).

## Discussion

Present results suggest that the capacity of each ovary to compensate ovarian functions (CO and COH) as well as their ability for hormone release is different for the right and left ovaries and depend on the age of the animal when ULO is performed.

The results also suggest that the ovaries' vagus nerve innervations modulate hormone secretion in an asymmetric way, and the hormone secretion is related to both, which ovary remains in situ, and the age of the animals when ULO treatment is performed.

Puberty onset is a multi-factorial regulated process that involves changes in serum hormone levels, maturation of the hypothalamus-pituitary-ovary axis, environmental conditions, and ovarian innervation [8,9,17,29,30]. Bilateral vagotomy in pre-pubertal female rats results in lower estradiol serum levels than in control animals [8], and in a decrease in the *in vitro *ovarian steroidogenic activity [9]. Then, the low estradiol serum levels observed in vagotomized 24-days old rats could explain the delay in the onset of puberty.

The results presented herein show that unilateral vagotomy followed by unilateral ovariectomy results in lower estradiol serum concentrations and in a delay in the age of puberty onset. Vagotomy, without ovariectomy, did not have an apparent effect on the age of puberty onset. Compared to control animals, right-ULO to adult rats results in lower ovulation rates, while ovulation rates were not affected when the left ovary was extirpated [28]. On the other hand, changes in ovulation rate were not observed in pre-pubertal animals with ULO, suggesting that the physiologic response to a missing ovary depends on the age in which the animal is treated (pre-pubertal or adult). In pre-pubertal rats, unilateral or bilateral vagotomy did not modify ovulation rates [8]. In adult rats, left vagotomy to animals with the left ovary in situ resulted in ovulation rates increases, while a reverse response was observed in rats with right vagotomy and the right ovary in situ [28]. Present results suggest that in pre-pubertal rats the vagus nerve modulates ovulatory responses in a stimulatory way, since right ULO to rats with the left vagotomy resulted in a lower number of ovulating animals.

There is evidence that in the adult rat the CO does not depend on the *in-situ *ovary [28]; similar to the results in pre-pubertal rats presented herein.

Compared to ULO rats, no difference in CO was observed in ULO adult rats submitted to right vagotomy and left ovary extirpation, [28]. Present results show that the same treatment (right vagotomy and right ovary in situ) in pre-pubertal animals results in an increase of CO. Such differences in the response between adult and pre-pubertal rats could reflect the lack of maturation of the CNS's regulatory mechanisms.

There is evidence of multiple neural pathways connecting the ovaries with the CNS. At present, the participation of the vagus nerve, the superior ovarian nerve (SON), and the ovarian plexus in such mechanisms is supported by several experimental evidences [6-8,12,17,22,23]. Also, that the SON and the ovarian plexus act through their connection to the celiac-mesenteric ganglion [31-33]. Based on the effects of bilaterally vagotomy to adult rats with ULO, Burden and Lawrence [1,4] proposed that vagal ovarian innervation stimulates COH, independently of the extirpated ovary. Trkulja and Lackovic, [27] showed that bilateral vagotomy blocked COH when performed immediately after ULO, and had no apparent effects when vagotomy was performed four and a half hours after ovary extirpation. The results obtained in the present study suggest that COH is modulated asymmetrically during puberty, suggesting that COH modulation is carried out by neural information traveling to the CNS through the vagus nerve and other neural pathways and depends on the animal's age.

Several studies have shown that the neural information originating from each ovary is asymmetric [33,34]. Toth et al 2007 [35] provided evidence that more neurons are involved in the supraspinal innervation of the left ovary than of the right. According to Klein and Burden [31], there is a slight asymmetry in the population of true-blue labeled neurons from the superior ovarian nerve and the ovarian plexus nerve, with the right ovary receiving more input from a greater number of the cells in the celiac superior-mesencephalic ganglia (CSMG) than the left ovary, besides the observed differences were not statistically significant.

Previously, we showed that compared to cyclic sham-operated animals, acute extirpation of the left or right ovary results in changes in estradiol, progesterone and testosterone serum levels, and that these changes depend on the hormone studied and the day of the cycle when surgery was performed; indicating an asymmetric ability for hormone release by the ovaries [36-38]. Gerendai et al. [39] did not observe such effects when performing ULO to adult rats nine days after surgery. Butcher [40] observed that the effects on progesterone levels resulting from ULO performed on diestrus1-day depend on the time elapsed between surgery and hormone measurements: progesterone concentrations in ULO animals were significantly lower 11-hours after surgery, while no significant differences were observed in animals sacrificed 30-hours after ULO.

Present results suggest that, on the day of first vaginal estrus the right ovary has a higher capacity to secrete progesterone than the left one. Such effect is opposite to previous observations in adult rats treated the day of estrus [36]. Such differences could be explained by the time elapsed between surgery and autopsy (one hour and 10 days), the age of the treated animals and the ability of the system to compensate the lack of one ovary.

Several researchers have shown that the vagal innervation stimulates the release of progesterone secretion. *In vitro *studies with ovaries obtained from pre-pubertal vagotomized rats show that the ovaries of treated animals secrete lower concentrations of progesterone than ovaries from control animals [9]. Abdominal vagotomy to pregnant rats lowers plasma progesterone levels, with a concurrent reduction in ovarian 3β-hydroxisteroide deshydrogenase activity [41]. A similar response occurred in pre-pubertal animals when bilateral vagotomy was performed to 24-days old rats, or unilateral or bilateral vagotomy was performed to 28-day old rats [8]. The results obtained in the present study indicate that in ULO rats, the lack of vagal innervation to the in situ ovary results in higher progesterone serum levels, suggesting that the vagal innervation plays an inhibitory role on progesterone secretion. The participation of the adrenal glands in such progesterone serum levels increase cannot be excluded, since its contribution varies along the estrous cycle and depends on the cholinergic system [37].

It has been shown that the ability to secrete testosterone by the right and left testis and ovaries of adult rats is different [42,43]. Present results suggest that neural information carried by the left vagus nerve of 28-day -old animals plays an inhibitory role on testosterone secretion, while the right vagus nerve plays a stimulatory role.

Unilateral or bilateral sectioning of the vagus nerve to pre-pubertal rats results in lower estradiol serum levels [8]. Because present results show that ULO performed to rats with ipsilateral sectioning of the vagus nerve results in significantly lower estradiol serum levels, we suppose that the compensatory mechanisms regulating estradiol secretion are not fully developed in the pre-pubertal rat.

## Conclusion

based on present and previous results, we postulate that in the pre-pubertal rat with ULO, the participation of the vagus nerve in regulating the secretion of steroidal hormones is asymmetric, depends on the vagal innervation, and varies with the age of the animals when treatment is performed.

## References

[B1] Burden HW, Lawrence (1977). The Effect of denervation on compensatory ovarian hypertrophy. Neuroendocrinology.

[B2] Burden HW, Lawrence, Hodson C (1980). Effect of abdominal vagotomy of the pregnant rat on pituitary content of prolactin and gonadotropins. IRCS Med Sci.

[B3] Burden HW, Leonard MJ, Smith C, Lawrence (1983). The sensory innervation of the ovary: A horseradish peroxidase study in the rat. Anat Rec.

[B4] Burden HW, Leonard MJ, Hotson CA, Louis TM, Lawrence (1986). Effect of abdominal vagotomy at proestrus on ovarian weight, ovarian antral follicles, and serum levels of gonadotropins, estradiol and testosterone in the rat. Neuroendocrinology.

[B5] Gabella G, Pease HL (1973). Number of axon in the abdominal vagus of the rat. Brain Res.

[B6] Gerendai I, Tóth IE, Boldogköi Z, Medveczky I, Halász B (2000). CNS structures presumably involved in vagal control of ovarian function. J Auton Nerv Syst.

[B7] Gerendai I, Kocsis K, Halász B (2002). Supraspinal connections of the ovary: Structural and functional aspects. Micr Res Tech.

[B8] Morales L, Betanzos R, Domínguez R (2004). Unilateral or bilateral vagotomy performed on prepubertal rats at puberty onset of female rat deregulates ovarian function. Arch Med Res.

[B9] Ojeda SR, White SS, Aguado LI, Advis JP, Andersen JM (1983). Abdominal vagotomy delays the onset of puberty and inhibits ovarian function in the female rat. Neuroendocrinology.

[B10] Gerendai I, Tóth IE, Boldogkoi Z, Medveezky I, Halász B (1998). Neural labeling in the rat brain and spinal cord from the ovary using viral transneuronal tracing technique. Neuroendocrinology.

[B11] Edgren RA, Parlow AF, Peterson DL, Jones RC (1965). On the mechanism of ovarian hypertrophy following hemicastration in rats. Endocrinology.

[B12] Gerendai I, Halász B (1997). Neuroendocrine Asymmetry. Front Neuroendocrinol.

[B13] Peppler RD, Greenwald GS (1970). Influence of unilateral ovariectomy on follicular development in cycling rats. Am J Anat.

[B14] Hirshfield AN (1983). Compensatory Ovarian hypertrophy in the long-term hemicastrate rat: size distribution of growing and atretic follicles. Biol Reprod.

[B15] Otani T, Sasamoto S (1982). Plasma and pituitary hormone changes and follicular development after unilateral ovariectomy in cyclic rats. J Reprod Fertil.

[B16] Flores A, Morales L, Ulloa-Aguirre, Domínguez R (1990). Acute changes in serum levels of luteinising hormone and follicle stimulating hormone, ovulation and follicular growth induced by stress, unilateral ovariectomy or mechanical stimulation of the ovarian pedicle at different stages of the oestrous cycle of the rat. Med Sci Res.

[B17] Aguado LI, Ojeda SR (1984). Prepubertal ovarian function is finely regulated by direct adrenergic influences. Role of noradrenergic innervation. Endocrinology.

[B18] Burden HW, Ben-Jonathan N, Barh JM, Weiner RI (1985). The adrenergic innervation of mammalian ovaries. Catecholamines as Hormone Regulator.

[B19] Chávez R, Domínguez R (1994). Participation of the superior ovarian nerve in the regulation of compensatory ovarian hypertrophy: the effects of its section performed on each day of the oestrous cycle. Endocrinology.

[B20] Gerendai I, Marchetti B, Maugery S, Amico RM, Scapagnini U (1978). Prevention of compensatory ovarian hypertrophy by local treatment of the ovary with 6-OHDA. Neuroendocrinology.

[B21] Morales L, Chávez R, Domínguez R (1993). Participation of the superior ovarian nerve in the regulation of ovulation in the prepubertad rat: differential effects of unilateral and bilateral section of the nerve. Med Sci Res.

[B22] Morales L, Chávez R, Ayala ME, Domínguez R (1998). Effects of unilateral or bilateral superior ovarian nerve section in prepubertad rats on the ovulatory response to gonadotrophin administration. J Endocrinol.

[B23] Morán C, Morales L, Razo RS, Apolonio J, Quiróz U, Chavira R, Domínguez R (2003). Effects of sensorial denervation induced by capsaicin injection at birth or on day three of life, on puberty, induced ovulation and pregnancy. Life Sci.

[B24] Nakamura Y, Kato H, Terranova PF (1992). Abdominal vagotomy decreased the number of ova shed and serum progesterone levels on estrus in the cyclic hamster. Endocrinol Jap.

[B25] Chávez R, Sánchez S, Ulloa-Aguirre A, Domínguez R (1989). Effects on Oestrus cyclity and ovulation of unilateral section of the vagus nerve performed on different days of the oestrus cycle in the rat. J Endocrinol.

[B26] Burden HW, Lawrence IE, Louis TM, Hodson CA (1981). Effects of abdominal vagotomy on the estrous cycle of the rat and the induction of pseudopregnancy. Neuroendocrinology.

[B27] Trkulja V, Lackovic Z (2001). Vagal influence on compensatory ovarian grown is important only briefly after hemicastration. Exp Biol Med (Maywood).

[B28] Chávez R, Cruz ME, Domínguez R (1987). Differences in the ovulation rate of the right or left ovary in unilaterally ovariectomized rats: effect of ipsi- and contralateral vagus nerves on the remaining ovary. J Endocrinol.

[B29] Foster LD, Ebling FJ, Knobil E, Neill J (1999). Puberty, in monoprimate mammals. The Physiology of Reproduction.

[B30] Ramaley JA (1980). *Biological Clocks and puberty onset*. Fed Proc.

[B31] Klein C, Burden HW (1988). Anatomical localization of afferent and postganglionic sympathetic neurons innervating the rat ovary. Neurosci Lett.

[B32] Klein C, Ray RH, Burden HW (1989). Direct electrical stimulation of the superior ovarian nerve in rats causes an increase in neuronal activity in the ipsilateral ovarian plexus nerve. Brain Res.

[B33] Morán C, Franco A, Morán JL, Handal A, Morales L, Domínguez R (2005). Neural activity between ovaries and the prevertebral celiac-superior mesenteric ganglia varies during the estrous cycle of the rat. Endocrine.

[B34] Domínguez R, Morales L, Cruz ME (2003). Ovarian Asymmetry. Ann Rev Biomed Sci.

[B35] Tóth IE, Wiesel O, Boldogköi Z, Balint K, Tapaszti Z, Gerendai I Predominance of supraspinal innervation of the left ovary. Microsc Res Tech.

[B36] Barco AI, Flores A, Chavira R, Damián-Matsumura P, Domínguez R, Cruz ME (2003). Asymmetric effects of acute hemiovariectomy on steroid hormone secretion by the in situ ovary. Endocrine.

[B37] Flores A, Meléndez G, Palafox MT, Rodríguez JO, Barco AI, Chavira R, Domínguez R, Cruz ME (2005). The participation of the cholinergic system in regulating progesterone secretion through the ovarian-adrenal crosstalk varies along the estrous cycle. Endocrine.

[B38] Cruz ME, Chávez R, Domínguez R (1986). Ovulation follicular growth and ovarian reactivity to exogenous gonadotropins in adult rats with unilateral or bilateral section of the vagi nerves. Rev Investig Clín.

[B39] Gerendai I, Csaba Zs, Voko Z, Csernus V (1995). Involvement of a direct neural mechanism in the control of gonadal function. J Steroid Biochem Mol Biol.

[B40] Butcher RL (1977). Changes in gonadotropins and steroids associated with unilateral ovariectomy of the rat. Endocrinology.

[B41] Lawrence IE, Burden HW, Louis TM (1978). Effect of abdominal vagotomy of thr pregnant rat on LH and progesterone concentration and fetal resorption. J Reprod Fertil.

[B42] Frankel AI, Chapman JC, Cook B (1989). Testes are asymmetric in the testicular hemicastration response of the male rat. Journal Endocrinology.

[B43] Flores A, Rodríguez JO, Palafox MT, Meléndez G, Barco A, Chavira R, Cruz ME, Domínguez R (2006). The acute asymmetric effects of hemiovariectomy on testosterone secretion vary along the estrous cycle. The participation of the cholinergic system. Reprod Biol Endocrinol.

